# Maternal health equity in Georgia: a Delphi consensus approach to definition and research priorities

**DOI:** 10.1186/s12889-023-15395-3

**Published:** 2023-03-30

**Authors:** Natalie D. Hernandez, Angela D. Aina, L. Joy Baker, Sarah C. Blake, Alexis B. Dunn Amore, Cheryl G. Franklin, Zsakeba T. Henderson, Michael R. Kramer, Fleda Mask Jackson, Elizabeth Mosley, Lauren Nunally, Shirley Sylvester

**Affiliations:** 1 Center for Maternal Health Equity, Department of Community Health and Preventive Medicine, Morehouse School of Medicine, 30310 Atlanta, GA USA; 2 Black Mamas Matter Alliance, Atlanta, GA USA; 3 Wellstar West Georgia Medical Center, LaGrange, GA USA; 4 Department of Health Policy & Management, Rollins School of Public Health, Emory University, Atlanta, GA USA; 5 Nell Hodgson Woodruff School of Nursing, Emory University, Atlanta, GA USA; 6 Department of Obstetrics and Gynecology, Morehouse School of Medicine, Atlanta, GA USA; 7 March of Dimes, Arlington, VA USA; 8 Department of Epidemiology, Rollins School of Public Health, Emory University, Atlanta, GA USA; 9 MAJAICA, LLC, Atlanta, GA USA; 10 Georgia State University School of Public Health, Atlanta, GA USA; 11 Georgia Perinatal Quality Collaborative, Duluth, GA USA; 12 Johnson & Johnson, Women’s Health, Office of the Chief Medical Officer, New Brunswick, NJ USA

**Keywords:** Delphi, Maternal health, Equity, Definition, Disparities, Social determinants of health, Perinatal, Georgia, Pregnancy, Maternal mortality

## Abstract

**Background:**

Pregnancy-related mortality in the United States is the greatest among all high-income countries, and Georgia has one of the highest maternal mortality rates—almost twice the national rate. Furthermore, inequities exist in rates of pregnancy-related deaths. In Georgia, non-Hispanic Black women are nearly 3 times more likely to die from pregnancy-related complications than non-Hispanic White women. Unlike *health equity*, a clear definition of *maternal health equity* is lacking, overall and in Georgia specifically, but is needed to reach consensus and align stakeholders for action. Therefore, we used a modified Delphi method to define maternal health equity in Georgia and to determine research priorities based on gaps in understanding of maternal health in Georgia.

**Methods:**

Thirteen expert members of the Georgia Maternal Health Research for Action Steering Committee (GMHRA-SC) participated in an iterative, consensus-driven, modified Delphi study comprised of 3 rounds of anonymous surveys. In round 1 (web-based survey), experts generated open-ended concepts of maternal health equity and listed research priorities. In rounds 2 (web-based meeting) and 3 (web-based survey), the definition and research priorities suggested during round 1 were categorized into concepts for ranking based on relevance, importance, and feasibility. Final concepts were subjected to a conventional content analysis to identify general themes.

**Results:**

The consensus definition of maternal health equity created after undergoing the Delphi method is: maternal health equity is the ultimate goal and ongoing process of ensuring optimal perinatal experiences and outcomes for everyone as the result of practices and policies free of interpersonal or structural bias that tackle current and historical injustices, including social, structural, and political determinants of health impacting the perinatal period and life course. This definition highlights addressing the current and historical injustices manifested in the social determinants of health, and the structural and political structures that impact the perinatal experience.

**Conclusion:**

The maternal health equity definition and identified research priorities will guide the GMHRA-SC and the broader maternal health community for research, practice, and advocacy in Georgia.

**Supplementary Information:**

The online version contains supplementary material available at 10.1186/s12889-023-15395-3.

## Introduction

Over the last 3 decades in the United States, risk factors and complications associated with poor pregnancy outcomes—diabetes mellitus, maternal age ≥ 40 years, chronic hypertension, hypertensive disorders of pregnancy, and ≥ 5 live births—have increased substantially (by 261%, 194%, 182%, 149%, and 33%, respectively) [1]. Pregnancy-related mortality in the United States is the greatest among all high-income countries [2–4] and—compared with non-Hispanic White women—risk of pregnancy-related death is > 3 times among non-Hispanic Black women and more than double among American Indian and Alaska Native women [2,5]. Based on data from 14 states (2008–2017), leading causes of pregnancy-related deaths are cardiomyopathy (13.9%) and cardiovascular conditions (13.9%) among non-Hispanic Black women, and mental health conditions (14.9%), cardiovascular conditions (13.4%), and hemorrhage (13.4%) among non-Hispanic White women [6].

Within the United States, Georgia has one of the highest maternal mortality rates—almost twice the national rate (50.8 vs. 29.6 deaths/100,000 live births in 2019) [7, 8]. Mortality rates vary based on timing of death, being higher during pregnancy or within 42 days of pregnancy end (34.1 vs. 20.2 deaths/100,000 live births, respectively) than after day 42 through less than 1 year of pregnancy end (16.7 vs. 9.4 deaths/100,000 live births, respectively). This disparity is further worsened by stark racial-ethnic disparities in Georgia and in the United States at large.

According to US Census data (as of July 1, 2022), 76% of the population are White alone (59% non-Hispanic or Latino), 14% are Black or African American alone, and 19% are Hispanic or Latino [9]. In Georgia, 59% of the population are White alone (51% non-Hispanic or Latino), 33% are Black or African American alone, and 10% are Hispanic or Latino. Similar to national statistics, non-Hispanic Black women in Georgia are substantially more likely to die from pregnancy-related complications than non-Hispanic White women. Of the 64 pregnancy-related deaths in Georgia in 2019, 43 were non-Hispanic or Latino Black or African American women (98.4 deaths/100,000 live births) and 16 were non-Hispanic or Latino White women (29.2 deaths/100,000 live births) [7, 8]. Moreover, 76 of the 159 counties in Georgia did not have a single obstetrics/gynecology provider in 2017-2018 [10]. According to the March of Dimes Maternity Care Deserts Report, [11] nearly 4 in 10 counties in Georgia are either considered “maternity care deserts” (counties in which access to maternity health care services is limited or absent, either through lack of services or barriers to a woman’s ability to access that care) or to have little to no access to maternity care. Research and advocacy groups, most notably the Black Mamas Matter Alliance [12], have spearheaded efforts to reduce maternal morbidity and mortality among Black women by using human rights, reproductive justice, and birth justice frameworks, and positioning maternal health (i.e., the health of women during pregnancy, childbirth, and the postnatal period) as a human rights issue.

The maternal mortality crisis in Georgia was formally recognized by the state in 2019, resulting in the House of Representatives Study Committee on Maternal Mortality, and its corresponding findings and recommendations [13]. However, significant gaps persist in our understanding of why women in Georgia have negative maternal health experiences. *Health equity* has been defined globally in various ways, with clear and common themes such as optimal health for all people, valuing all populations equally, meeting all health needs, removing structural influences on health and healthcare, and addressing inequities in health outcomes [25–30]. However, a clear definition of *maternal health equity* is lacking, overall and in Georgia specifically, but is needed to reach consensus and align stakeholders for action.

In 2020, the Morehouse School of Medicine Center for Maternal Health Equity and Johnson & Johnson initiated a partnership to address maternal health disparities in Georgia and established the Georgia Maternal Health Research for Action Steering Committee (GMHRA-SC). The goal of GMHRA-SC is to assemble researchers, clinicians, policy experts, and community leaders to align on an inclusive, actionable, sustainable, and scalable evidence-based approach to improve maternal health outcomes, especially for Black mothers, in Georgia. Two initial aims were to create a definition of maternal health equity in Georgia and determine corresponding research priorities. To that end, we used a modified Delphi method to reach expert consensus on these topics.

## Methods

### Panel selection

Researchers, health care providers, community-based health advocates, and policy experts across a range of disciplines with knowledge about maternal health equity and/or those involved in current or past studies addressing Black maternal health in Georgia were purposefully selected as experts to be part of the GMHRA-SC and participate in this study. Experts were identified using searches of Google, Google Scholar, PubMed, and NIH RePORTER (keywords: maternal health, equity, Black women, and research); personal networks; a list of indivudals who expressed interest in the GMHRA-SC; and faculty listings from applicable departments at universities across Georgia. Potential experts also nominated other individuals for consideration. These varied approaches were used to mitigate introduction of selection bias.

Potential experts were ranked on the basis of qualifications and inclusion criteria, which comprised maternal health expertise, specifically with racial/ethnic populations; health equity expertise; research in the area of maternal health, women’s health, and health equity; and individuals who led organizations focused on maternal health/women’s health. A panel of 13 experts were invited via e-mail to participate in the GMHRA-SC. Inclusion criteria and expectations of participation were described in the invitation e-mail. Finally, participants were asked for their commitment to be part of the GMHRA-SC and its ongoing research, including this study.

### Delphi approach

From December 2020 to February 2021, the GMHRA-SC conducted an iterative, 3-step, consensus-driven, modified Delphi study to: (1) define maternal health equity in Georgia and (2) determine research priorities based on identified gaps in current understanding of maternal health in the state. A modified Delphi approach was used to gain consensus on a subject for which opinions and definitions vary and guidance is lacking. Our approach comprised a qualitative assessment (round 1), a ranking evaluation (round 2), and consensus development (round 3). In contrast to the original Delphi method [14–17], panel members were known to one another and interacted with one another after the third round to reach consensus on the definition of maternal health equity in Georgia. This step allowed for further clarification and the opportunity to present arguments to justify viewpoints. In prior studies, while the original Delphi method produced the most accurate results (vs. face-to-face meetings or a nominal group technique), group interactions in the latter were perceived as highly cooperative and effective [18].

**Fig. 1:**
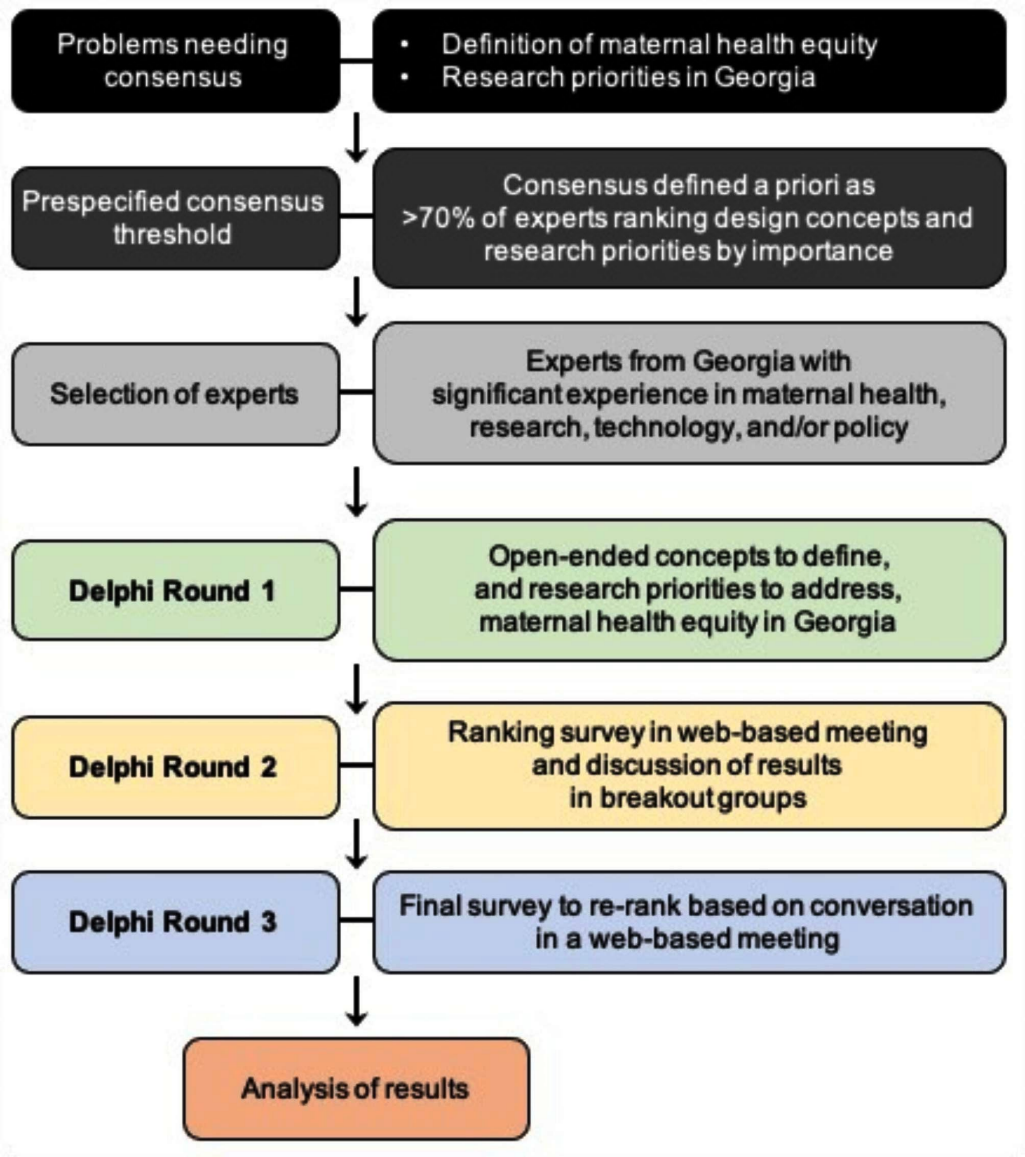
Modified Delphi method. A modified Delphi approach was used to define maternal health equity in Georgia and to determine research priorities based on gaps in understanding of maternal health in Georgia

Three rounds of anonymous surveys (considered optimal to reach consensus [14–17]) were hosted on SurveyMonkey.com and employed to enable adequate reflection of group responses. For round 1, an email with a link to the survey was sent to GMHRA-SC members (Fig. 1). Three open-ended questions were asked: (1) “How do you define maternal health equity?”, (2) “What are the most important research priorities to address maternal health equity in Georgia?”, and (3) “How can we ensure that research design and/or program implementation for maternal health takes an equity-informed approach?” (**Supplementary Table 1**).

In rounds 2 and 3, GMHRA-SC members ranked concepts identified in the prior round, taking into account relevance, importance, and feasibility (Fig. 1). Each question had a follow-up question, where experts could elaborate or explain responses, as well as indicate if anything was missed, if any input was misinterpreted, or if anything beyond the choices provided should be considered in the next round or final outcomes.

The round-2 survey was conducted during a web-based GMHRA-SC meeting (Fig. 1), where experts independently completed the survey. GMHRA-SC members were asked: (1) “Noting that all of [ the following] are important to improve maternal health, what needs to be included in a definition of maternal health equity for Georgia?” and (2) “What are the most important research priorities to address maternal health equity in Georgia?” (Supplementary Table 1). For question 1, experts ranked definition concepts relative to one another. For question 2, experts categorized research priorities independently of one another as unimportant, important but not urgent, or important and urgent. After survey results were compiled, experts were assigned to 1 of 2 breakout groups, were provided the results, and were asked to participate in a Nominal Group Technique [19]—a tool for gathering group opinion where participants generate ideas silently and independently of each other, and then discuss and evaluate the ideas as a group. GMHRA-SC members accessed and completed the round-3 survey (**Supplementary Table 1**), during which they were asked the same questions as in round 2, and re-ranked definition concepts and re-categorized research priorities (Fig. 1).

### Data analysis

Descriptive statistics were used for participants’ demographic characteristics and group responses to each question in all 3 rounds. Consensus was defined a priori as > 70% of experts ranking design concepts and research priorities by importance. A project team, responsible for collating and analyzing results, comprised 2 coders and 2 project leads (NDH and SS).

The project team collected the qualitative responses from round 1, and NDH and SS collaborated using an inductive content analysis procedure [20, 21] to arrive at a consensus on the components of the definition of maternal health equity in Georgia to be considered in the second round. For round 2, the project team collated survey data and performed calculations (question 1, mean rankings; question 2, n [%]), analyzed survey results, considered responses from the breakout sessions, and modified concepts accordingly for use in the third round. For round 3, the project team collated survey data and performed calculations, analyzed survey results, and generated a final list of concepts. Finally, the 2 coders and NDH independently identified themes that emerged across the entire list of concepts using an inductive content analysis procedure [20, 21]. Consensus was reached on a set of statements that were combined to create a final definition of maternal health equity in Georgia.

## Results

### Demographic characteristics of experts

All 13 experts participated and were surveyed. All but 1 expert identified as female (92%), and the majority identified as Black (69%). More than half of experts held a doctoral degree (54%) and worked in an academic setting (54%). The experts represented a variety of public health and clinical health care fields, with one-third representing public health (33%; **Supplementary Table 2**).

### Open-ended concepts of maternal health equity and research priorities in Georgia

In round 1, 12 experts (1 expert was unavailable) provided their views on the definition of maternal health equity in Georgia (**Supplementary Fig. 1**). Experts’ responses focused on several important concepts, including health disparities, social determinants of health, racial and socioeconomic equity, equitable clinical care, structural bias, and individual needs. Results from round 1 yielded several concepts for the definition of maternal health equity and research priorities for Georgia. The top 2 research topics were determining the (1) *causes of, and solutions to, racial inequity in maternal health outcomes and services*, and (2) *policy-related factors that impede access to high-quality maternal health services*.

### Definition of maternal health equity

Responses on the definition of maternal health equity from round 1 were categorized into 9 concepts, which were provided to the experts for ranking relative to one another using values ranging from 1 to 9 in the second round (Table 1). Although 13 experts were expected to respond in the second round, 14 anonymous responses were received. Because responses were anonymous, all 14 responses were considered. Two concepts with the lowest rankings (highest mean scores) in round 2 were (1) *include pregnant people of all genders* [mean score, 8.57 of possible 9.00] and (2) *approaches health from a life course perspective* [6.71]). Although these 2 concepts received the lowest rankings, experts agreed during the breakout session that they represented important components of maternal health equity and should be incorporated into other broader concepts. Therefore, these 2 concepts were combined with other definition concepts to create 7 definition concepts for re-ranking in round 3 (Table 1).

**Table 1:** Ranking of Maternal Health Equity Definition (Rounds 2 and 3)

#	Definition of Maternal Health Equity^a^	Mean Ranking Score	
		**Round 2,** ranked 1–9^**b **^**(n = 14)**^c^	**Round 3, ** ranked 1–7^**b **^**(n = 12)**^d^
1	Eliminating underlying drivers (advantages and disadvantages between groups) of disparities *across the life course*^e^	4.00	2.92
2	Tackling current and historical injustices manifested in the social determinants of health and the structural and political structures that impact the perinatal experience	3.64	2.00
3	All mothers *and birthing people of all genders*^f^ have equal opportunity to have a positive perinatal experience and achieve well-being	2.36	4.00
4	Appropriate and high-quality clinical care services for each mother’s *and birthing person’s*^g^ individual needs	5.00	3.75
5	Care is free from interpersonal or structural bias	3.93	4.33
6	Include pregnant people of all genders	8.57	N/A
7	Access to safe services	5.79	5.33
8	Access to affordable care	5.00	5.67
9	Approaches health from a life course perspective	6.71	N/A

Ranking of each statement was relative to one another

^a^Question to experts: Noting that all of these are important to improve maternal health, what needs to be included in a definition of maternal health equity for Georgia?

^b^One (1) represented the highest priority

^c^Includes 1 additional response, which was included because its origin could not be determined due to the anonymity of responses

^d^One expert was unavailable

^e^The phrase “across the life course” was added for round 3 after removing concept #9

^f^The phrase “and birthing people of all genders” was added for round 3 after removing concept #6

^g^The phrase “and birthing person’s” was included only in round 3

N/A, not applicable because this question was not included in round 3

In round 3, experts ranked the 7 definition concepts relative to one another using values ranging from 1 to 7. Two concepts of maternal health equity that received the highest rankings (lowest mean scores) were (1) *tackling current and historical injustices manifested in the social determinants of health and the structural and political structures that affect perinatal experience* (mean score, 2.00 of possible 7.00) and (2) *eliminating underlying drivers of disparities across the life course* (2.92) (Table 1). The 2 concepts with the lowest rankings (highest mean scores) were (1) *access to affordable care* (5.67 of possible 7.00) and (2) *access to safe services* (5.33).

**Fig. 2:**
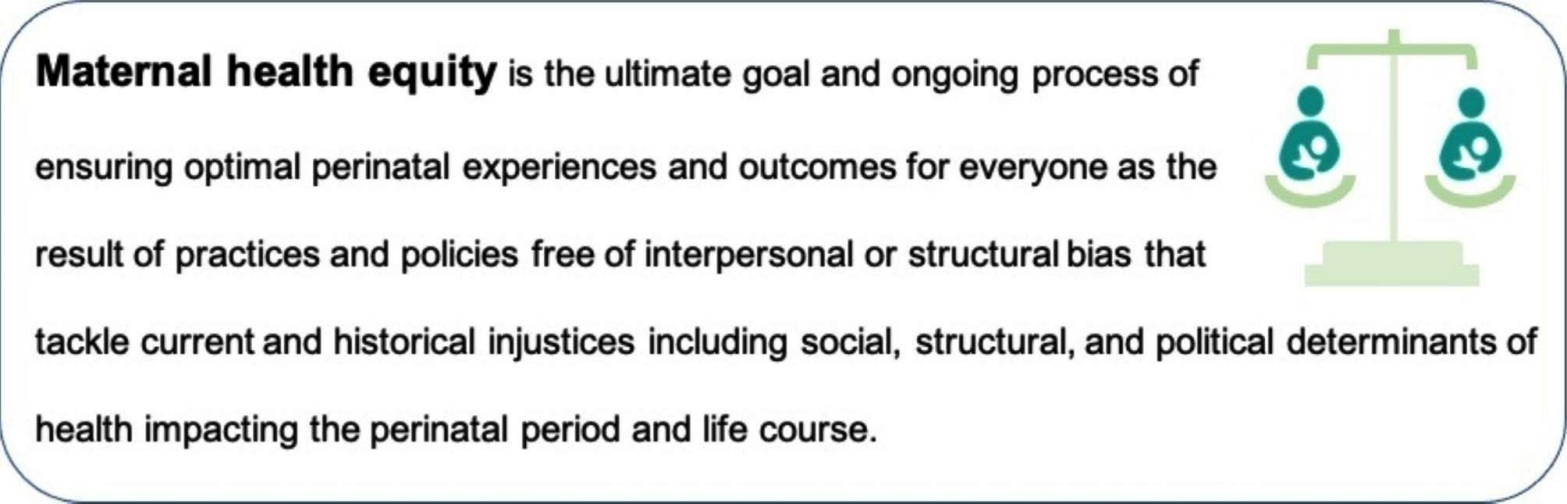
Consensus definition of maternal health equity in Georgia

Using compiled findings from the 3 rounds, a definition of maternal health equity was created that highlights the need to address current and historical injustices in social determinants of health, and the structural and political biases that adversely affect the perinatal experience (Fig. 2).

### Research priorities in Georgia

Based on experts’ responses in round 1, 15 research priorities (14 specific and 1 nonspecific [*other*]) were developed and provided to the experts in round 2 for categorization (Table 2). No expert provided a response for the nonspecific [*other*] research priorities in the second round; therefore, this research priority was omitted from round 3. Most experts considered 2 research priorities, (1) *understand and measure the social, structural, and political drivers of maternal health disparities* and (2) *causes of, and solutions to, racial inequity in maternal health outcomes and services*, important and urgent in the second round. These 2 research priorities were considered related and were combined into a single concept—*understand and measure the causes of, and solutions to, social, structural, and political drivers of maternal health disparities*—for the third round.

**Table 2:** Ranking of Research Priorities in Georgia (Rounds 2 and 3)

Research Priorities to Address Maternal Health Equity in Georgia, n (%)^a^	Round 2 (n = 14)^b^			Round 3 (n = 12)^c^		
	UI	I not U	I and U	UI	I not U	I and U
Understand and measure the *causes of, and solutions to*,^d^ social, structural, and political drivers of maternal health disparities	0 (0)	3 (21)	11 (79)	0 (0)	3 (25)	9 (75)
Causes of, and solutions to, racial inequity in maternal health outcomes and services^e^	0 (0)	1 (8)	12 (92)	N/A		
Evaluating barriers to *and benefits*^f^*of* full-spectrum care, including midwives, doulas, postpartum care, and abortion services	0 (0)	4 (29)	10 (71)	0 (0)	6 (50)	6 (50)
Understanding maternal mental health^d^	0 (0)	5 (38)	8 (62)	0 (0)	4 (33)	8 (67)
Evaluating what’s worked in reducing disparities and how to translate results to Georgia	0 (0)	3 (21)	11 (79)	0 (0)	0 (0)	12 (100)
Evaluating Black women’s *and birthing people’s nonclinical*^g^ experience of maternal care services	0 (0)	4 (29)	10 (71)	1 (8)	6 (50)	5 (42)
Factors that contribute to patient distrust of providers	0 (0)	8 (57)	6 (43)	0 (0)	9 (75)	3 (25)
Understanding patients’ *clinical and nonclinical*^h^ expectations of their care	0 (0)	7 (50)	7 (50)	1 (8)	6 (50)	5 (42)
Uncovering the interactions of challenges and assets within Black communities and families and how they produce risks and resilience for maternal and birth outcomes^i^	0 (0)	7 (50)	7 (50)	0 (0)	6 (55)	5 (45)
Patient-centered maternal care, including provider-level factors, that impede that care	0 (0)	3 (21)	11 (79)	1 (8)	4 (33)	7 (58)
Effects of stress *and sleep health*^j^ on maternal morbidity	0 (0)	10 (71)	4 (29)	0 (0)	3 (25)	9 (75)
Protective effects of *safe, affordable, and accessible*^k^ radical care models/intensive care coordination and social supports	0 (0)	7 (50)	7 (50)	0 (0)	6 (50)	6 (50)
Policy-related factors that impede access to high-quality maternal health services^e^	0 (0)	1 (8)	12 (92)	0 (0)	2 (17)	10 (83)
Life course factors that contribute to higher risk of poor maternal health outcomes, particularly for Black and low-income mothers	0 (0)	5 (36)	9 (64)	0 (0)	5 (42)	7 (58)
Other (please specify)	NR			N/A		

Ranking of each statement was independent of one another

^a^Question to experts: What are the most important research priorities to address maternal health equity in Georgia?

^b^Includes 1 anonymous respondent during a live meeting who inadvertently responded likely not knowing that they were not requested to provide feedback

^c^One expert was unavailable

^d^The phrase “causes of, and solutions to,” was included only in round 3

^e^n=13 in round 2

^f^The phrase “and benefits of” was included only in round 3

^g^The phrase “and birthing people’s nonclinical” was included only in round 3

^h^The phrase “clinical and nonclinical” was included only in round 3

^i^n=11 in round 3

^j^The phrase “and sleep health” was included only in round 2

^k^The phrase “safe, affordable, and accessible” was included only in round 3

I, important; N/A, not applicable as this question was not included in round 3; NR, no response; U, urgent; UI, unimportant

The 13 research priorities from round 2 were provided to the experts for re-categorization (independently of one another) in round 3 as unimportant, important but not urgent, or important and urgent (Table 2). At the end of round 3, most (≥ 75%) of the 12 experts who responded to the survey considered the following research priorities in Georgia important and urgent: (1) *evaluating what’s worked in reducing disparities and how to translate results to Georgia* (12 experts [100%]), (2) *policy-related factors that impede access to high-quality maternal health services* (10 [83%]), (3) *understand and measure the causes of, and solutions to, social, structural, and political drivers of maternal health disparities* (9 [75%]), and (4) *effects of stress on maternal morbidity* (9 [75%]).

## Discussion

The purpose of this modified Delphi exercise was to collaboratively generate a definition of maternal health equity and research priorities in Georgia that were both accurate and actionable. A consensus was reached after 3 rounds of anonymous surveys of 13 experts in maternal health equity. The definition included concepts (e.g., structural and social determinants of health, health disparity, racial and socioeconomic equity, and equitable clinical care) that the experts considered relevant to maternal health equity in Georgia. More than 75% of the experts also reached consensus on several research priorities, including (1) assessing measures that are known to reduce disparities and how they could be implemented in Georgia; (2) identifying policy-related barriers to accessing high-quality maternal health services; (3) understanding the causes of social, structural, and political drivers of maternal health disparities, and how they can be improved; and (4) determining how stress might play a role in maternal morbidity.

The Delphi method is a form of consensus exploration used to understand a range of opinions from a panel of experts on a particular topic, including whether consensus exists and the extent of consensus [14, 22, 23]. Experts are asked for anonymous input on a set of questions, and the responses are processed by a moderator. Results are shared with the experts, who then are asked another set of similar or different questions. The method requires anonymity of responses and provision of feedback after each round of questions. Delphi methodology can be modified and adapted to fit specific needs and context. Defining maternal health equity and determining research priorities in Georgia are particularly suited to Delphi methodology, which purposefully and systematically includes stakeholders, because of the diversity of caregivers, complexity of the factors that influence the pregnancy journey, and the dynamic state of healthcare delivery.

Defining maternal health equity is important for understanding and addressing maternal morbidity and mortality because definitions drive measurements and constrain, illuminate, and shape the questions we ask and/or how we ask them. Definitions also shape answers and how we interpret them. If equity is the goal, the act of defining and the voices leading the definition of maternal health equity are central to affecting change. Including diverse voices at the table and centering Black women in the definition-making process are key to capturing all important facets of maternal health equity. Social determinants of health, which have a critical impact on maternal health outcomes, need to be an important part of the definition [24].

Formal definitions of maternal health equity are lacking, overall and in the peer-reviewed literature specifically. Health equity has been defined in various ways, including as: (1) assurance of the conditions for optimal health for all people by valuing all populations equally, recognizing and rectifying historical injustices, and providing resources according to need [25, 26] (2) an approach to comprehensively meet people’s reproductive and sexual health needs, with explicit attention to structural influences on health and health care and grounded in a desire to achieve the highest level of health for all people and address inequities in health outcomes [27]; (3) attainment of the highest level of health for all people, which requires valuing everyone equally with focused and ongoing societal efforts to address avoidable inequalities, historical and contemporary injustices, and the elimination of health and health care disparities [28]; (4) creating equal opportunities for health and bringing health differentials down to the lowest level possible [29]; and (5) a fair and just opportunity for everyone to be as healthy as possible, which requires removing obstacles to health such as poverty, discrimination, and their consequences, including powerlessness and lack of access to good jobs with fair pay, quality education and housing, safe environments, and health care [30]. *Maternal* health equity, in contrast, has not been clearly defined. Instead, authors identify risk factors for maternal health inequities, discuss issues of equity and justice in the context of specific patient populations, and/or offer potential mechanisms to improve maternal health and reduce inequities [25, 31–33].

Potential factors underlying the high maternal mortality rates in the United States include chronic medical conditions, such as hypertension and diabetes mellitus, disadvantageous socioeconomic conditions, inadequate access to health care, and race. However, elements that focus on individuals and specific risk factors fail to identify the fundamental causes of maternal health inequities in the United States, including systemic, historical, and structural biases. “Structural determinants of health” and “root causes of inequities” are significant components that drive the social determinants of maternal health, and some experts hypothesized that maternal health inequities in the United States are ultimately the result of structural racism [25, 34]. In another, racism, along with other structural inequities such as gender oppression, classism, and economic deprivation—especially in Black and Indigenous populations—have received limited attention as core factors that influence individual behaviors [25, 27, 33].

Our definition of maternal health equity expands upon existing definitions of health equity, social determinants of health, and root causes of health by focusing solely on the health of pregnant and birthing people and the perinatal experience. This definition, and the research priorities identified, highlight specific actions that can begin to uncover and address the fundamental causes of poor maternal health outcomes in Georgia and illuminate policy strategies that can sustain maternal health equity in the state. Our next step will be a vote to adopt the definition for the GMHRA.

Communities, funders, payors, and policy makers share a critical role in reducing and ultimately eliminating maternal health inequities in the United States. At the community level, promising models for the much-needed change exist. Some measures that could help improve maternal health outcomes and reduce costs—especially for people who are most likely to experience inferior maternal outcomes—include fair compensation through Medicaid for community-based doulas and birth centers, simplification of the doula licensure process, and deliberate efforts to train racially and culturally diverse obstetricians, nurse midwives, and doulas [35]. Improvements in the perinatal workforce also should include championing more people of color to own or lead birth centers by unlocking access to resources and capital. Bray and colleagues identified the myth of the default standard of Whiteness as a key cause of maternal morbidity and mortality among Black birthing people [36]. To dismantle this myth, the authors proposed several actions, including: (1) placing Black women at the center of discussions and decisions about Black maternal health; (2) including knowledge and experiences of Black communities in curricula for medical education and training; (3) including community-generated big data in the research with, for, and by Black women; (4) providing socially, culturally, and racially concordant clinical care; and (5) targeted investments in Black health care providers, students, researchers, educators, and communities at large.

Our work was intentionally focused on the state of Georgia, which has one of the highest maternal mortality rates in the United States. However, this study sets an example for other states to adopt a similar approach and identify research priorities specific to their at-risk population(s). Although research priorities may be different in other states/regions because of differences in health care and/or social, racial, and structural conditions, some of the themes we identified likely are common between Georgia and other states, especially Southern states. Ultimately, the definition of maternal health equity uncovered here could serve as the North Star to guide efforts in other states affected by inequities in maternity services.

### Study limitations

The Delphi approach has some inherent limitations [17]. Because the approach typically requires considerable input from participants, response rates can be low, especially as the number of surveys increases over time [37]. However, response rates from experts in the present study, which included 3 surveys to reach consensus, were high: 12/13 experts provided responses in all 3 rounds. The number of participants in our study was relatively small, which may have made responses uneven and introduced bias, especially because participants were not selected randomly—they were carefully selected based on location, research interests, and expertise. Unfortunately, despite the broad use of Delphi methodology, guidance on what constitutes consensus is lacking [15, 16]. For the present study, we prespecified a relatively high cutoff threshold (70%) to define consensus. Although inclusion of all possible concepts to generate consensus is unrealistic, efforts were made to include any concepts the experts considered important. For example, because experts agreed that the 2 lowest-ranking concepts for the definition of maternal health equity in the second round (*include pregnant people of all genders* and *include approaches health from a life course perspective*) were important, they were combined with other broader concepts for the third round.

### Conclusions and future directions

A consensus definition for maternal health equity highlights the current and historical injustices manifested in the social, structural, and political determinants of health that adversely affect the perinatal and life-course experiences of pregnant and birthing people, as well as their children, families, and communities. The definition and identified research priorities to address maternal health equity will guide the future work of the GMHRA-SC and broader maternal health community in terms of research, practice, and advocacy in Georgia, which can be a model for other states. This work builds on and hopes to strengthen the historical and ongoing research and advocacy by community-led reproductive justice groups, including Black Mamas Matter Alliance and SisterSong.

## Electronic supplementary material

Below is the link to the electronic supplementary material.

Supplementary Material 1

## Data Availability

All data generated and analyzed are contained within the manuscript or supplementary information.

## Declarations

### Research involving humans and/or the use of human tissue samples or human data

Not applicable: no experiments on humans and/or use of human tissue samples or human data; a modified Delphi approach was used to achieve consensus among experts/authors.

## List of Abbreviations

***GMHRA-SC***: Georgia Maternal Health Research for Action Steering Committee
