# Supplementary material for: Maternal health equity in Georgia: a Delphi consensus approach to definition and research priorities
Source: BMC Public Health. 2023 Mar 30;23:596. doi: 10.1186/s12889-023-15395-3 (PMC10061967; doi:10.1186/s12889-023-15395-3)
Supplement: Supplementary file 1 — Supplementary Material 1 [file 12889_2023_15395_MOESM1_ESM.pdf]

**Supplementary Table 1.** Survey questions

**Round 1: open-ended questions**

1. How do you define maternal health equity?
2. What are the most important research priorities to address maternal health equity in Georgia?
3. How can we ensure research design and/or program implementation for maternal health takes an equity-informed approach?

**Round 2: closed-ended questions**

1. Noting that all of these are important to improve maternal health, what needs to be included in a definition of maternal health equity for Georgia? Rank 1-9, with 1 being the highest priority to include in the definition. (You can click and drag the options into your preferred order)*
    - Eliminating underlying drivers (advantages and disadvantages between groups) of disparities
    - Tackling current and historical injustices manifested in the social determinants of health, structural, and political structures that impact the perinatal experience
    - All mothers have equal opportunity to have a positive perinatal experience and achieve wellbeing
    - Appropriate and high-quality clinical care services for each mother's individual needs
    - Care is free from interpersonal or structural bias
    - Include pregnant people of all genders
    - Access to safe services
    - Access to affordable care
    - Approaches health from a life course perspective

2. What are the most important research priorities to address maternal health equity in Georgia?* Response options: unimportant, important but not urgent, or important and urgent
    - Understand and measure the social, structural, and political drivers of maternal health disparities
    - Causes of, and solutions to, racial inequity in maternal health outcomes and services
    - Evaluating barriers to full-spectrum care, including midwives, doulas, postpartum care, and abortion services
    - Understanding maternal mental health
    - Evaluating what's worked in reducing disparities and how to translate results to Georgia
    - Evaluating Black women's experience of maternal care services

- Factors that contribute to patient distrust of providers
- Understanding patients' expectations of their care
- Uncovering the interactions of challenges and assets within Black communities and families and how they produce risks and resilience for maternal and birth outcomes
- Patient-centered maternal care including provider-level factors that impede that care
- Effects of stress and sleep health on maternal morbidity
- Protective effects of radical care models/intensive care coordination and social supports
- Policy-related factors that impede access to high-quality maternal health services
- Life course factors that contribute to higher risk of poor maternal health outcomes, particularly for Black and low-income mothers
- Other (please specify)

**Round 3: closed-ended questions**

1. Noting that all of these are important to improve maternal health, what needs to be included in a definition of maternal health equity for Georgia? Rank 1-7, with 1 being the highest priority to include in the definition. (You can click and drag the options into your preferred order)*
    - Eliminating underlying drivers (advantages and disadvantages between groups) of disparities across the life course
    - Tackling current and historical injustices manifested in the social determinants of health and the structural and political structures that impact the perinatal experience
    - All mothers and birthing people of all genders have equal opportunity to have a positive perinatal experience and achieve wellbeing
    - Appropriate and high-quality clinical care services for each mother and birthing person's individual needs
    - Care is free from interpersonal or structural bias
    - Access to safe services
    - Access to affordable care

2. What are the most important research priorities to address maternal health equity in Georgia?* Response options: unimportant, important but not urgent, or important and urgent
    - Understand and measure the causes of, and solutions to, social, structural, and political drivers of maternal health disparities

- Evaluating barriers to and benefits of full-spectrum care, including midwives, doulas, postpartum care, and abortion services
- Understanding maternal mental health
- Evaluating what's worked in reducing disparities and how to translate results to Georgia
- Evaluating Black women's and birthing people's non-clinical experience of maternal care services
- Factors that contribute to patient distrust of providers
- Understanding patients' clinical and non-clinical expectations of their care
- Uncovering the interactions of challenges and assets within Black communities and families and how they produce risks and resilience for maternal and birth outcomes
- Patient-centered maternal care including provider-level factors that impede that care
- Effects of stress on maternal morbidity
- Protective effects of safe, affordable, and accessible radical care models/intensive care coordination and social supports
- Policy-related factors that impede access to high-quality maternal health services
- Life course factors that contribute to higher risk of poor maternal health outcomes, particularly for Black and low-income mothers

*Participants also were asked: Please include any feedback (e.g., is something missing? are categories misrepresented?)

**Supplementary Table 2.** Demographic Characteristics of Experts

| **Characteristic, n (%)** | **Experts (N=13)** |
|---|---|
| Female sex | 12 (92%) |
| Race/ethnicity | |
| Black/African American | 9 (69%) |
| White | 3 (23%) |
| Hispanic | 1 (8%) |
| Degree | |
| PhD | 7 (54%) |
| MD | 4 (31%) |
| MPH | 2 (15%) |
| Setting | |
| Academic | 7 (54%) |
| Clinical | 3 (23%) |
| Health advocacy | 2 (15%) |
| Industry | 1 (8%) |
| Focus | |
| Public health | 4 (31%) |
| Obstetrician/gynecologist | 3 (23%) |
| Nurse/midwife/doula | 3 (23%) |
| Research/technology | 3 (23%) |

MD, doctor of medicine; MPH, master of public health; PhD, doctor of philosophy

**Supplementary Table 3.** Individual responses in round 2 for ranking 9 concepts for potential inclusion in the definition of maternal health equity for Georgia.

Question: Noting that all of these are important to improve maternal health, what needs to be included in a definition of maternal health equity for Georgia? Rank 1-9, with 1 being the highest priority to include in the definition.

| Concepts to potentially include in the definition of maternal health equity | Rank from 1 (highest priority) to 9 (lowest priority), n (%) | | | | | | | | | n[a] | Mean ranking score |
|---|---|---|---|---|---|---|---|---|---|---|---|
| | 1 | 2 | 3 | 4 | 5 | 6 | 7 | 8 | 9 | | |
| Eliminating underlying drivers (advantages and disadvantages between groups) of disparities | 1 (7.1) | 5 (35.7) | 2 (14.3) | 1 (7.1) | 0 (0.0) | 2 (14.3) | 1 (7.1) | 2 (14.3) | 0 (0.0) | 14 | 4.00 |
| Tackling current and historical injustices manifested in the social determinants of health, structural, and political structures that impact the perinatal experience | 4 (28.6) | 4 (28.6) | 0 (0.0) | 0 (0.0) | 2 (14.3) | 1 (7.1) | 1 (7.1) | 2 (14.3) | 0 (0.0) | 14 | 3.64 |
| All mothers have equal opportunity to have a positive perinatal experience and achieve wellbeing | 8 (57.1) | 2 (14.3) | 0 (0.0) | 2 (14.3) | 0 (0.0) | 1 (7.1) | 1 (7.1) | 0 (0.0) | 0 (0.0) | 14 | 2.36 |
| Appropriate and high-quality clinical care services for each mother's individual needs | 0 (0.0) | 1 (7.1) | 3 (21.4) | 1 (7.1) | 3 (21.4) | 2 (14.3) | 4 (28.6) | 0 (0.0) | 0 (0.0) | 14 | 5.00 |
| Care is free from interpersonal or structural bias | 0 (0.0) | 2 (14.3) | 2 (14.3) | 6 (42.9) | 3 (21.4) | 1 (7.1) | 0 (0.0) | 0 (0.0) | 0 (0.0) | 14 | 3.93 |
| Include pregnant people of all genders | 0 (0.0) | 0 (0.0) | 0 (0.0) | 0 (0.0) | 0 (0.0) | 0 (0.0) | 2 (14.3) | 2 (14.3) | 10 (71.4) | 14 | 8.57 |
| Access to safe services | 1 (7.1) | 0 (0.0) | 1 (7.1) | 2 (14.3) | 2 (14.3) | 2 (14.3) | 2 (14.3) | 3 (21.4) | 1 (7.1) | 14 | 5.79 |
| Access to affordable care | 0 (0.0) | 0 (0.0) | 5 (35.7) | 1 (7.1) | 2 (14.3) | 2 (14.3) | 3 (21.4) | 1 (7.1) | 0 (0.0) | 14 | 5.00 |
| Approaches health from a life course perspective | 0 (0.0) | 0 (0.0) | 1 (7.1) | 1 (7.1) | 2 (14.3) | 3 (21.4) | 0 (0.0) | 4 (28.6) | 3 (21.4) | 14 | 6.71 |

[a]Includes 1 additional response, which was included because its origin could not be determined due to the anonymity of responses.

**Supplementary Table 4.** Individual responses in round 3 for ranking 7 concepts for potential inclusion in the definition of maternal health equity for Georgia.

| Question: Noting that all of these are important to improve maternal health, what needs to be included in a definition of maternal health equity for Georgia? Rank 1-7, with 1 being the highest priority to include in the definition. | | | | | | | | | |
|---|---|---|---|---|---|---|---|---|---|
| **Concepts to potentially include in the definition of maternal health equity** | **Rank from 1 (highest priority) to 7 (lowest priority), n (%)** | | | | | | | n | Mean ranking score |
| | 1 | 2 | 3 | 4 | 5 | 6 | 7 | | |
| Eliminating underlying drivers (advantages and disadvantages between groups) of disparities across the life course | 3 (25.0) | 5 (41.7) | 0 (0.0) | 1 (8.3) | 0 (0.0) | 3 (25.0) | 0 (0.0) | 12 | 2.92 |
| Tackling current and historical injustices manifested in the social determinants of health, structural, and political structures that impact the perinatal experience | 6 (50.0) | 3 (25.0) | 2 (16.7) | 0 (0.0) | 0 (0.0) | 1 (8.3) | 0 (0.0) | 12 | 2.00 |
| All mothers and birthing people of all genders have equal opportunity to have a positive perinatal experience and achieve wellbeing | 2 (16.7) | 1 (8.3) | 3 (25.0) | 1 (8.3) | 2 (16.7) | 0 (0.0) | 3 (25.0) | 12 | 4.00 |
| Appropriate and high-quality clinical care services for each mother's and birthing person's individual needs | 0 (0.0) | 3 (25.0) | 2 (16.7) | 5 (41.7) | 0 (0.0) | 1 (8.3) | 1 (8.3) | 12 | 3.75 |
| Care is free from interpersonal or structural bias | 1 (8.3) | 0 (0.0) | 2 (16.7) | 2 (16.7) | 6 (50.0) | 0 (0.0) | 1 (8.3) | 12 | 4.33 |
| Access to safe services | 0 (0.0) | 0 (0.0) | 1 (8.3) | 2 (16.7) | 3 (25.0) | 4 (33.3) | 2 (16.7) | 12 | 5.33 |
| Access to affordable care | 0 (0.0) | 0 (0.0) | 2 (16.7) | 1 (8.3) | 1 (8.3) | 3 (25.0) | 5 (41.7) | 12 | 5.67 |

**Supplementary Figure 1.** Open-ended concepts of maternal health equity from 12 experts[a] (round 1)

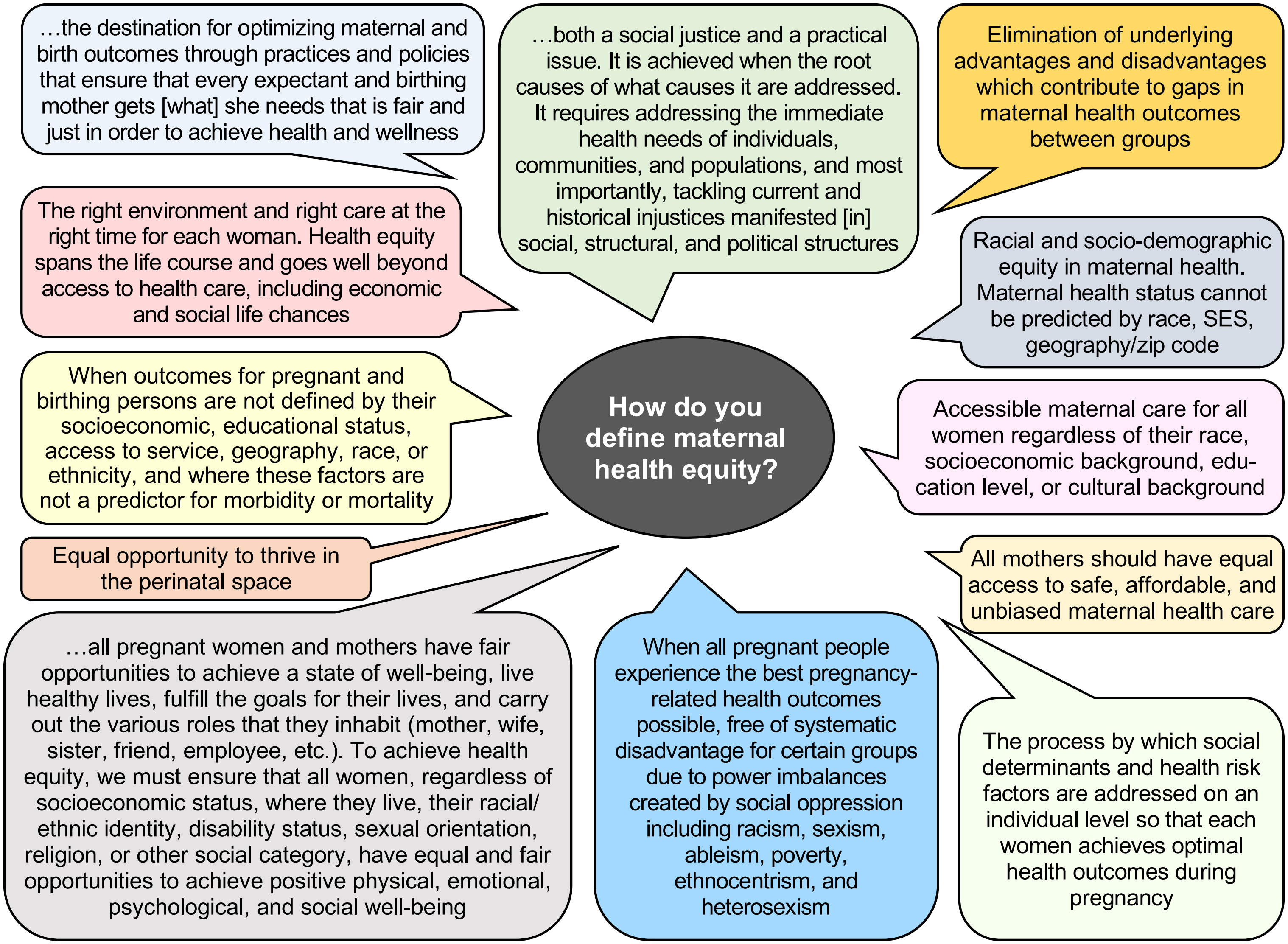


[a]One expert was unavailable.
